# Use of Envelope Domain III Protein for the Detection of IgG Type Antibodies Specific to Zika Virus by Indirect ELISA

**DOI:** 10.3390/diagnostics13030462

**Published:** 2023-01-26

**Authors:** Oumar Ndiaye, Cheikh Tidiane Diagne, Ahmed Abd El Wahed, Fatou Dia, Moussa Dia, Adama Faye, Silvania Da Veiga Leal, Menilita dos Santos, Maria da Luz de Lima Mendonça, Carolina Cardoso da Silva Leite, Cheikh Saad Bouh Boye, Juliet E. Bryant, Philippe Desprès, Ousmane Faye, Amadou Alpha Sall, Oumar Faye

**Affiliations:** 1 Virology Department, Institut Pasteur de Dakar, Dakar BP 220, Senegal; 2 Institut de Santé et Développement (ISED), Université Cheikh Anta DIOP de Dakar, Dakar BP 16390, Senegal; 3 Institute of Animal Hygiene and Veterinary Public Health, University of Leipzig, D-04103 Leipzig, Germany; 4 National Institute of Public Health, Largo do Desastre da Assistência, Chã de Areia, Praia P.O. Box 719, Cape Verde; 5 World Health Organization, Country Office—AFRO Prédio Comum das Nações Unidas Meio Achada S. António 1° Andar, Praia P.O. Box 266, Cape Verde; 6 Centre de Reference IST/VIH, Centre Hospitalier Universitaire Aristide Le Dantec, Dakar BP 3001, Senegal; 7 The Global Fund Against AIDS, Tuberculosis and Malaria, Chemin du Pommier 40, 1218 Le Brand-Sacconex, Switzerland; 8 Processus Infectieux en Milieu Insulaire Tropical (PIMIT), Université de La Réunion, INSERM UMR 1187, CNRS 9192, IRD 249, Plateforme CYROI, 97490 Sainte-Clotilde, France

**Keywords:** Zika, diagnostics, ELISA, recombinant, antigen, envelope, IgG

## Abstract

Zika virus (ZIKV) diagnostics are crucial for proper antenatal and postnatal care and also for surveillance and serosurvey studies. Since the viremia during ZIKV infection is fleeting, serological testing is highly valuable to inform diagnosis. However, current serology tests using whole virus antigens frequently suffer from cross reactivity issues, delays, and technical complexity, especially in low and middle income countries (LMICs) and endemic countries. Here, we describe an indirect ELISA to detect specific IgG antibodies using the ZIKV envelope domain III (EDIII) protein expressed in Drosophila S2 cells as an immunogen. Using a total of 367 clinical samples, we showed that the EDIII-ELISA was able to detect IgG antibodies against ZIKV with high sensitivity of 100.0% and specificity of 94.7% when compared to plaque reduction neutralization tests (PRNTs) as the gold standard and using 0.208 as the cut-off OD value. These results show the usefulness of the recombinant envelope domain III as an alternative to standard whole virus proteins for ZIKV diagnostics as it improves the sensitivity and specificity of IgG ELISA assay when used as an immunogen. This method should, therefore, be extended to serological diagnostic techniques for other members of the flavivirus genus and for use in IgM diagnostic testing.

## 1. Introduction

Zika virus (ZIKV) is an emerging arthropod borne virus transmitted by a number of mosquito species [1] belonging to the genus *flavivirus* of the family *Flaviviridae*. The first ZIKV outbreak was confirmed in Yapp Island in Micronesia in 2007, and throughout the last decade, it has been responsible of a number of outbreaks throughout the world [2]. The virus came to public attention in 2015–2016 when a dramatic increase in cases of Guillain Barré in adults and microcephaly cases in neonates in Brazil was linked to a large-scale outbreak of ZIKV [3,4]. As for the other flaviviruses to which ZIKV is closely related such as dengue virus (DENV), yellow fever virus (YFV), and West Nile virus (WNV), the clinical presentation of ZIKV is not specific and can be easily confused with other arboviruses [5]; therefore, specific laboratory diagnostics are required to correctly distinguish between the infectious agents and to provide adequate clinical management [6].

During ZIKV infection, viremia generally lasts less than 7 days and direct virus detection can be accomplished using reverse transcription-polymerase chain reaction (RT-PCR) or virus isolation during the acute phase [7]; however, as viremia is fleeting and resolves usually within the first week of illness, current laboratory diagnostics for ZIKV are mainly based on indirect methods aiming to detect IgM and IgG class antibodies [8]. However, the biggest challenge of serological assays is the cross-reactivity of ZIKV antibodies with other flaviviruses, especially with dengue virus [9]. It has been proven that vaccination or past infections with other flaviviruses considerably increases the level of cross reactivity, rendering interpretation of serological results very difficult, especially in flavivirus endemic regions [10]. In order to bolster the specificity of results, plaque reduction neutralization test (PRNT) has been introduced to confirm positive ELISA results [11]; however, this method is currently difficult to implement in low and middle income countries (LMICs) as it requires manipulations of the live virus in cell culture, and therefore, can only be performed in specialized biosafety level 3 (BSL3) containment laboratories by highly trained personnel. These requirements generate long turnaround times that can reach 5 days before obtaining confirmations. Under certain rare circumstances, PRNT testing can also be affected by cross reactivity between flaviviruses [9]. Given these technical hurdles, most laboratories rely on ELISA methods that are principally based on whole viral antigens produced from cell culture or suckling mouse brain antigen. The World Health Organization (WHO) has issued laboratory diagnostic algorithms recommending anti-ZIKV antibody-based testing in patients presenting 7+ days after symptom onset [12]. IgM-type antibodies are detectable as early as the 5th day of illness and can persist up to 6 months postinfection, and IgG-type antibodies are detectable from the 10th day of the disease and can theoretically persist for years [13].

Indirect IgG ELISA is used on samples >10 days postonset of symptoms and gives better results if paired samples are collected 2–3 weeks apart [12]. Whole viral particles exhibiting the surface envelope protein have very similar epitopes and, therefore, yield cross reactive responses [14]. To circumvent the cross reactivity issue of these whole viral particles, in recent years, it has been proposed to use alternative surface-presenting viral proteins that may afford increased specificity. In the flavivirus genus, the structural proteins E (envelope), the prM (membrane precursor) protein along with the nonstructural secreted protein NS1 represent the major targets of the host antibody immune response [15]. Most of the neutralizing antibodies that are generated are directed against the envelope protein. The envelope protein comprises 3 domains, domain I (EDI), domain II (EDII), and domain III (EDIII), with most of the immunodominant epitopes present in the EDIII domain [16]. EDIII of the envelope protein has been found to be highly diverse among different flaviviruses; thus, it is specific enough to potentially eliminate or significantly decrease cross reactivity in assays for serodiagnosis of flaviviruses [16]. Recently, a variety of teams have demonstrated the usefulness of using the EDIII protein in the serological diagnostics of different flaviviruses [17,18] and for a potential vaccine platform [19]. In 2021, a team demonstrated the usefulness of the ZIKV EDIII immunogen produced in *E.coli* showing promising results in a competitive ELISA format [20].

In this paper, we describe the use of ZIKV recombinant EDIII protein produced in drosophila cell lines (S2 expression system) on an indirect ELISA format by evaluating its performance among a well-characterized cohort of positive and negative samples. Positive ZIKV IgG samples were collected during the ZIKV virus outbreak in Cape Verde 2015–2016 [21] and negative ZIKV IgG samples were collected within the WHO Collaborating Center (WHO-CC) on Arboviruses and Viral Hemorrhagic Fevers of Institut Pasteur de Dakar (IPD). The PRNT test was used as the “gold standard” for the calculation of the performances. After statistical analysis and determination of the ideal cut-off point, the test performance was calculated using study samples and standards.

## 2. Materials and Methods

### 2.1. Ethics Statement

This study was conducted according to the guidelines of the Declaration of Helsinki. We used residual samples collected as part of approved ongoing surveillance activities conducted by IPD, which is a WHO-CC for Arboviruses and Hemorrhagic Fever Reference and Research. All samples were deidentified before performing laboratory characterization and analyses.

### 2.2. Sample Descriptions

For the evaluation of sensitivity and specificity, a set of 367 serum samples were used. The sensitivity panel is composed of 179 samples that had been previously found positive for ZIKV antibodies by inhouse IgG ELISA and PRNT; those samples were collected from the Virology Laboratory at the Achadinha Health Center (Praia, Cape Verde) for ZIKV diagnosis. For specificity, 93 symptomatic samples from the virology laboratory of Praia that tested negative, and 95 samples of various conditions negative to ZIKV PRNT were also collected within the WHO-CC (Table 1).

### 2.3. Study Design

This study aimed to assess the improvement of ELISA IgG assay by using EDIII protein as antigen and testing it among well-characterized biobanked serum samples. All panel samples had been stored at −80 °C after previous virological testing. Positive ZIKV samples had been tested by inhouse IgG ELISA specific to ZIKV and later confirmed by PRNT test, those samples comprise the panel of sensitivity as explained above. In order to assess the specificity of the assay, samples previously tested IgG and PRNT positive to closely related flaviviruses (YFV, WNV, and DENV) were selected; they were confirmed negative to ZIKV IgG and PRNT also. To further assess other potential cross reactivates/interferences, Malaria PCR positive samples, rheumatoid factor positive, and Neg All ** samples were selected and tested negative to ZIKV IgG and PRNT prior to incorporation in the study.

### 2.4. Recombinant EDIII from ZIKV

After alignment of EDIII proteins of the south pacific PF-25013-18 strain (Genbank Accession number: KX369547) and the reference strain (MR766), it is of note that the EDIII of PF-25013-18 differs from MR766 by a single amino-acid change from alanine to valine at position 342 (cf. Table 2).

The DES expression system (Life Technologies, Carlsbad, CA, USA) was required for the production of the recombinant domain III from the E protein (rEDIII) of the epidemic South Pacific ZIKV strain PF-25013-18 in *Drosophila* S2 cells as previously described [22]. Briefly, a synthetic gene coding for ZIKV.rEDIII corresponding to the C terminus of the E ectodomain (residues 297 to 408) was cloned into the shuttle vector pMT/BiP/SNAP, a derived pMT/BiP/V5-HisA plasmid (Life Technologies, Carlsbad, CA, USA) in which the SNAP-tag sequence (Covalys BioSciences AG, Altendorf, Switzerland) had been inserted in frame with the insect BiP signal peptide. The resulting plasmid pMT/BiP/SNAP-ZIKV.EDIII was transfected into S2 cells to establish a stable cell line S2/SNAP-ZIKV.EDIII. The production and the purification of soluble secreted SNAP-rEDIII proteins from the stable S2/SNAP-ZIKV.EDIII cell line were performed as previously described [23]. A stock of highly purified SNAP-rEDIII (1 mg/mL) was used for the ELISA tests.

### 2.5. Cells and Viruses

Viral stocks had been previously prepared using reference standard strains of the IPD’s WHO CC. The respective strains are: 17D for YFV, Dengue 2 New Guinea C (NGC) for DENV, Eg101 for WNV, and Monkey Rhesus (MR766) for ZIKV. All strains were grown at 28 °C in *Aedes albopictus* cell lines (C6/36) during 4 days for YFV, WNV, and ZIKV, whereas for DENV, the harvest was performed after 8 days’ incubation at 28 °C. YFV, WNV, and ZIKV strains were later titrated on PS cells (Porcine Stable kidney cell line, American type Culture Collection, Manassas, VA, USA), while DENV was titrated in African green monkey (*Chlorocebus* species) kidney epithelial cells (Vero) (ATCC: CCL-81, American Type Culture Collection, Manassas, VA, USA). Infected PS cells were grown for 5 days at 37 °C in L15 (Leibovitz’s 15) medium (10% heat-inactivated fetal bovine serum [FBS], 1% penicillin-streptomycin, 0.05% amphotericin B [Fungizone] (GIBCO by Life Technologies; USA), whereas infected Vero cells were incubated for 7 days in the same media at 37 °C with 5% CO_2_ prior to harvest.

### 2.6. Antigens and Antibodies

Polyclonal mouse ascitic fluids used in this study were prepared after intraperitoneal inoculation of inactivated whole viral strains previously produced by cell culture or mouse brain. Adult Swiss webster mice were immunized following a specific timepoint with the viral strains along with TG180 murine sarcoma cells. Ascitic fluids were collected 42 days later by ventral puncture.

Mouse brain antigens were prepared after intracerebral inoculation of the live viral strains previously produced. The mouse brains were collected after around 6 days’ incubation time following symptoms (hind leg paralysis, sickness, lethargy, etc.) and homogenized by manual trituration in PBS 1X buffer. After initial clarification steps, they were inactivated with 0.3% β-propiolactone overnight at 4 °C. The preparations were then stored at −80 °C until further use.

### 2.7. Serologic Tests

- Inhouse Indirect IgG ELISA

All samples positive to flaviviruses used for the IgG ELISA were tested by inhouse indirect IgG ELISA as the screening method. For indirect IgG ELISA, standard ELISA plates (Immulon II 96-well microtiter plates; Dynatech laboratories, Inc., El Paso, TX, USA) were coated with 100 µL of inhouse-prepared mouse hyper immune ascitic fluids specific to either YFV, DENV, WNV, or ZIKV at 1/1000 dilution in phosphate buffer saline (PBS) solution at 0.135 M and coated overnight at 4 °C. After the initial four washing steps with 300 µL of wash buffer (PBS 1X-Tween20 0.05%), 100 µL of inhouse-prepared specific corresponding mouse brain antigens to either YFV, DENV, WNV, or ZIKV were diluted at 1/40 in dilution buffer (PBS 1X-Tween20-1% skimmed milk) and incubated at 37 °C for 1 h. After 1 h incubation time, plates were washed four times with 300 µL of wash buffer and 100 µL of samples, negative and positive controls were added at 1/100 dilution in dilution buffer. After 1 h incubation at 37 °C and four washing steps with 300 µL of wash buffer, 100 µL of goat anti-Human IgG horseradish peroxidase conjugated (Seracare, Milford, MA, USA) diluted at 1 µg/mL in dilution buffer was added to the plates. After 1 h incubation at 37 °C and subsequent washing steps with 300 µL of wash buffer, specific binding was revealed by addition of 100 µL of ready to use 3,3’,5,5’-Tetramethylbenzidine (TMB) (Catalog number T0440-100ML, Sigma-Aldrich, Saint Louis, MO, USA) and subsequently stopped using 100 µL of 2 N Sulfuric acid (H_2_SO_4_). Plates were later read on the spectrophotometer at 450 nm wavelength and 620 nm as passive reference. Sera were considered positive if the optical density was >0.20 above the negative sera average and the ratio (R) between the sample and the negative control was >2.

- Plaque reduction neutralization test (PRNT)

Flaviviruses ELISA IgG positive samples were analyzed for flavivirus neutralizing antibodies using PRNT as described in [24].

(a) PRNT testing for ZIKV, WNV, and YFV

Briefly, heat-inactivated sera samples with positive and negative controls were diluted twofold starting at 1:10 in L15 medium (Thermo Scientific, Waltham, MA, USA). Then, 100 µL of the diluted samples were mixed separately with equal volumes of medium containing 1000 PFU/mL (plaque forming units) of the reference strains. Virus/serum mixtures were then incubated for 1 h at 37 °C and later used to infect PS cell monolayers in 24-well plates. After 1 h incubation at 37 °C, cells were covered with 400 µL of L15 medium containing 3% FBS (fetal bovine serum) and 0.4% carboxymethyl cellulose (CMC) and incubated for 4 days. Coloration of plaques was performed using blue-black dye solution (1% g/mL black amido, 1.36% g/mL sodium acetate, and 6% mL/mL acetic acid) staining. Briefly, after the 4 days’ incubation time, plates were washed with 400 µL of PBS 1X and subsequently 400 µL of a solution of the blue-black dye solution was added to each well and incubated for at least 1 h. Plates were then washed with 400 µL of PBS 1X and left to dry under a biosafety cabinet for at least 1 h. Neutralizing antibody titers were determined using a 90% cut-off value and a sample was classified as positive if the titer was ≥1/20.

(b) FRNT testing for DENV

Neutralization testing with DENV was performed using a focus reduction neutralization assay (FRNT) as no lytic strains were available during the evaluation to perform a PRNT.

Briefly, heat-inactivated sera samples with positive and negative controls were diluted twofold starting at 1:10 in DMEM medium (Dulbecco’s Modified Eagle Medium). Then, 100 µL of the diluted samples were mixed separately with equal volumes of DMEM containing 800 FFU/mL (focus forming units) of the Dengue 2 NGC reference strain. Virus/serum mixtures were then incubated for 1 h at 37 °C and later used to infect Vero cell monolayers in 24-well plates. After 1 h incubation at 37 °C, cells were covered with 400 µL of DMEM medium containing 3% FBS (fetal bovine serum) and 0.6% carboxymethyl cellulose (CMC) and incubated for 6 days. Detection of dengue focus units was performed using aminoethylcarbazole (AEC) coloration. Briefly, after the 6 days’ incubation, each well of the plates was fixed with 1 ml of a 1:1 solution of methanol and acetone. After an incubation at −20 °C for 30 min, plates were washed with 400 µL of PBS 1X and blocked with 250 µL of PBS 1X-3%FBS solution. After 30 min gentle shaking, 400 µL of Dengue 2 virus specific mouse ascitic fluid diluted at 1/1000 in PBS 1X was added in each well. After 1 h incubation, the plates were washed with 400 µL of PBS 1X and subsequently, 250 µL of goat anti-mouse IgG HRP conjugated (Seracare, Milford, MA, USA) diluted in PBS 1X-FBS 3% at 1 µg/mL was added to the plates. After 1 h incubation, plates were washed with 400 µL PBS 1X, and 400 µL of prediluted aminoethylcarbazole solution (ENZO, Farmingdale, NY, USA) at 1:7 in specific buffer provided by the manufacturer was added to the wells. After a 1 h incubation, the plates were washed and left to dry under a biosafety cabinet for at least 1 h. Neutralizing antibody titers were determined using a 90% cut-off value and a sample was classified as positive if the titer was ≥1/20.

(c) Recombinant Domain III indirect IgG ELISA

A total of 100 µL of specific ZIKV EDIII antigen was immobilized through passive adsorption in PBS 1X Buffer at the optimal concentration of 0.5 µg/mL on a NUNC Maxisorp high-binding plate overnight at 4 °C. The next day, plates were washed 4 times in 300 µL of wash buffer (PBS 1X-Tween20 0.05%). Plates were then blocked with 200 µL of PBS1X-Tween20 0.05–5% skimmed milk to saturate unbound sites for one hour at 37 °C. Plates were later washed 4 times with 300 µL of wash buffer, to get rid of excess of proteins, then 100 µL of the ZIKV reference sera were diluted at 1/100 in dilution buffer (PBS 1X-Tween20 0.05–2% Skimmed milk) and added on the plate. Plates were later incubated for one hour at 37 °C and washed 4 times with 300 µL of wash buffer. Specific binding was detected by adding 100 µL of goat anti-Human IgG horseradish peroxidase conjugate (Seracare, Milford, MA, USA) diluted at a concentration of 1 µg/mL in dilution buffer. After 1 h incubation time and 4 washing steps with 300 µL of wash buffer, 100 µL of ready to use Tetramethylbenzidine (TMB) (Catalog number T0440-100ML, Sigma-Aldrich, Saint Louis, MO, USA) was added to each well; the plate was covered and incubated at room temperature for 5 min. The reaction was stopped using 100 µL of 2 N H_2_SO_4_ solution, and optical densities were read on the spectrophotometer at 450 nm wavelength with 620 nm as baseline. Optical densities were normalized by subtracting OD of blank wells prior to analysis.

### 2.8. Statistical Analysis

We used receiver operating characteristic (ROC) curve analysis to determine the cut-off values for the EDIII antigen in the ELISA assay. The ROC curve analysis was performed with the Life module of XLSTAT (Addinsoft, Paris, France) implemented in Microsoft Excel and was constructed with OD values of the positive versus negative samples. The diagnostic performance was evaluated by estimation of sensitivity, specificity, accuracy, likelihood ratios, and area under the curve (AUC). Confidence intervals (CIs) were defined with 95% confidence level (95% CI). Confidence intervals and *p* values were obtained using a procedure first given in Clopper and Pearson [25], and *p* < 0.05 values were considered significant.

## 3. Results

### 3.1. Determination of Diagnostic Value of the IgG ELISA EDIII

**Figure 1:**
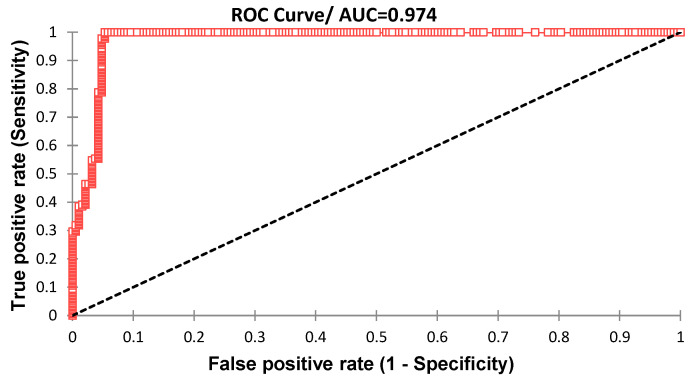
ROC curve of the EDIII IgG ELISA showing the evolution of sensitivity and false positive rate at each OD value.

In order to assess performance of the assay over the range of possible OD cut-off points, a ROC curve analysis was performed using the PRNT test as the gold standard on the classified positive and negative samples in Figure 1.

Table 3 summarizes the AUC values obtained from the ROC curve:

In order to identify the best cut-off value, the following graphs were traced and the diagnostic value calculated for each OD value.

**Figure 2:**
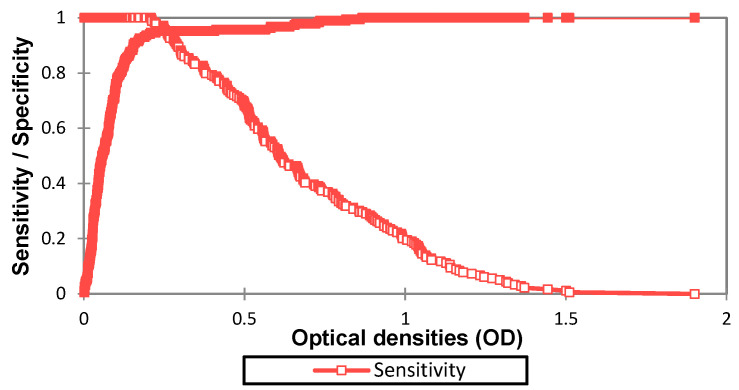
Sensitivity, specificity, TP, TN, FP, and FN per OD value.

**Figure 3:**
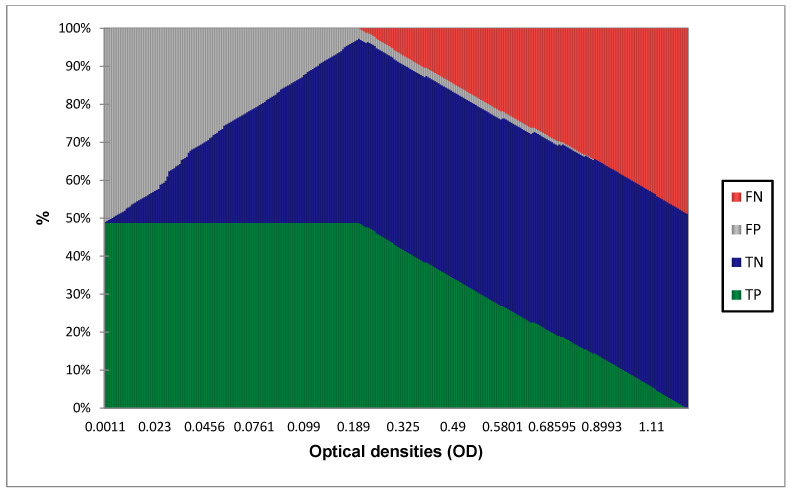
True positive (TP), true negative (TN), false positive (FP), and false negative (FN) proportions.

The results show that the AUC is at 0.974 which is superior to 0.9, this value corresponds to an outstanding discrimination between true positives (TP) and true negative samples (TN) with minimal false positive (FP) and no false negative (FN). Figure 2 and Figure 3 show that the best cut-off point is at 0.208, yielding 100% sensitivity [97.4–100] and 94.7% specificity [90.3–97.2]. In Table 4, the different performances at 0.208 cut-off are displayed.

### 3.2. Diagnostic Performance Calculation with 0.208 as Cut-Off Value

When considering performances per sample characterization (Table 5), the results show that specificity is higher among IgG WNV, POS Rheumatoid Factor, IgG CHIKV, Malaria PCR POS, and NEG all samples. The lowest value was found within the group of symptomatic IgG ZIKV Neg despite being at 91.4% [83.7–96.2]. All results are significant with *p* values < 0.05.

**Figure 4:**
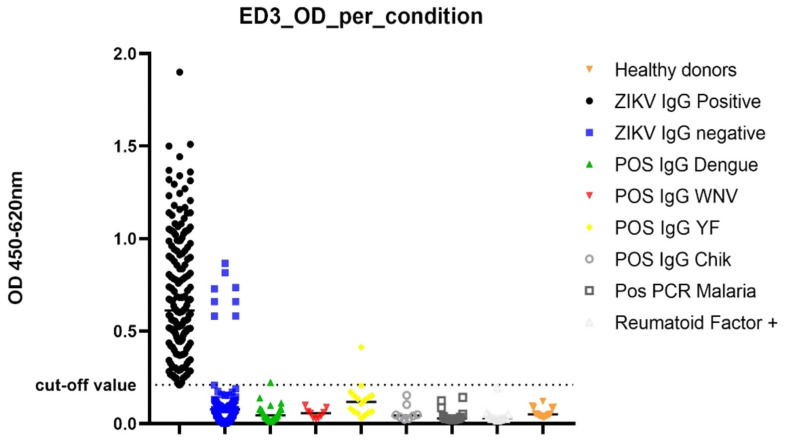
OD distribution per condition.

When comparing the optical density (ODs) values of each set of samples (Figure 4), values were significantly higher in ZIKV IgG positive samples compared to negative samples. False positive samples were reported among the cohort of symptomatic ZIKV IgG negative samples, Dengue IgG positive, and YFV IgG positives. Cross reactive sample ODs were higher in ZIKV IgG negative samples.

## 4. Discussion

Access and availability of specific diagnostic tests is crucial for the correct identification and confirmation of suspected cases of ZIKV infections. As infection during the first 3 months of pregnancy is often related to a higher risk of microcephaly in neonates [26], it is critical to ensure the accuracy and reliability of diagnostic tests to inform clinical case management during antenatal care. IgG antibodies tend to linger longer in the body than other infection biomarkers; they are, therefore, useful for assessing past infections [27]. However, cross reactivity and time constraints are recurrent problems in flavivirus diagnostics, hence most serological results are considered presumptive.

In this study, we investigated the potential role of the specific ZIKV EDIII protein expressed in eukaryotic cell lines as an immunogen during ELISA IgG diagnostics. We evaluated the diagnostic value of the rEDIII antigen on an indirect ELISA format using standard positive and negative ZIKV IgG samples collected from the ZIKV outbreak in Cape Verde in 2016 and additional well-characterized samples available through the WHO-CC of IPD. During the evaluation, the best cut-off value obtained was 0.208, which yielded a sensitivity of 100.0% [97.4–100.0] and specificity of 94.7% [90.3–97.2].

The EDIII IgG ELISA displayed a high sensitivity of 100.0%; this can be explained by the fact that most neutralizing antibodies are correlated to the rise in IgG antibody titers directed against the EDIII protein which is known to participate in receptor recognition [28]. EDIII also contains important linear antigenic epitopes that are the main target cell receptor-binding sites that assist viral entry into host cells [29]. This is verified when comparing the performance of other subunit antigen assays such as the NS1 ZIKA ELISA IgG [30] which gave a sensitivity between 71.0% to 88.0%, which is lower than what has been achieved with the ZIKV EDIII protein.

The test also displayed high specificity results of 94.7% compared to the PRNT test. This could be explained by the fact that EDIII is one of the most diverse proteins among flaviviruses [31]. This diversity might be explained by the role the EDIII protein plays in cellular recognition of both mosquito vectors and mammalian cell lines [28]. Even for closely related flaviviruses, the specificity of EDIII remains very high. DENV is known to yield a lot of cross reactions with ZIKV [32], but surprisingly, despite the fact that a large part of the population in Cape Verde was exposed to the DENV3 during the large scale outbreak of 2009 [33], only very few false positives were detected among the study samples representing this population; these findings suggest that the EDIII protein is sufficiently divergent between ZIKV and DENV to differentiate IgG antibodies elicited by the two viruses. Regarding other closely related flaviviruses such as YFV and WNV, and potentially interfering conditions such as rheumatoid factor and malaria, the results presented here indicate high specificities against those infections and conditions. The observed high specificity of the ZIKV EDIII suggests that this test could be used without need of a confirmatory test, even in areas where many flavivirus cocirculate. The lowest specificity obtained during the study was with ZIKV symptomatic IgG negative samples, with eight false positives detected, at 91.40% [83.75–96.21] when taking into consideration the PRNT as gold standard. Moreover, some of those samples did come out with relatively high ODs. We hypothesize that since the EDIII protein gave such high sensitivity, it might be more sensitive than the PRNT and ELISA IgG tests in detecting IgG antibodies among convalescent samples; when comparing the date postonset of symptoms of the different cross reactive samples, we found that they were all within 1 week postinfection. It is known that neutralization activity of antibodies is directly correlated to IgG maturation and the course of the disease [34]. Hence, early produced IgG antibodies with low avidity might not be picked up by the PRNT or inhouse ELISA test, whereas they may be detected by the EDIII IgG ELISA. Therefore, those samples could be true positive samples that could not be detected using standard IgG ELISA and PRNT.

The ZIKV strain used in the study was the lineage from the epidemic South Pacific strain, as the amino acid sequence of the EDIII is well conserved, and the divergence among the E proteins ranges between ~6% between lineages and ~2% within lineages [35]. The EDIII protein could, therefore, be used to test for samples of patients infected with East African or West African ZIKV lineages.

Globally, the sensitivity and specificity obtained showed a clear improvement of the performance of the ELISA test when using ZIKV EDIII protein as an immunogen. When compared to the PRNT test, the hands-on time is also far shorter as it takes around 4 h to obtain a result, whereas it takes 4 to 6 days when using PRNT. Despite being also highly sensitive and specific, PRNT testing does not distinguish between IgM or IgG antibodies in order to pose the diagnostics of recent or old ZIKV infection, contrary to the EDIII ELISA. The PRNT test is also expensive, labor intensive, and requires use of BSL3 lab and highly trained personnel, whereas the EDIII ELISA is cheap, easy to implement, and can be performed in a standard BSL2 lab.

When compared to the standard IgG ELISA, the advantage of the EDIII is the high sensitivity and specificity and, moreover, the fact that no mouse brain antigen is used in the assay which poses ethical problems due to the presence of animal tissue. Table 6 summarizes the advantages and drawbacks of the test compared to the standard IgG ELISA and PRNT test.

## 5. Conclusions

Both sensitivity and specificity results show that the EDIII IgG ELISA could be an ideal tool for serosurvey studies without need of confirmation by neutralization assay, but more importantly, it might be useful for the differential diagnostics of congenital infection by ZIKV during pregnancy or of neonatal cases with microcephaly. In preparation for future outbreaks and emergence events, it will be important to adapt the use of ZIKV EDIII antigen in IgM ELISA assays for the detection of acute cases or as a vaccine candidate.

## Figures and Tables

**Table 1:** Description of the specificity panel used for the evaluation.

**Specificity Panel**	
Characterizations	*n* =
**Related flaviviruses**	
POS IgG DENV	23
POS IgG WNV	9
POS IgG YF	16
**Alphavirus**	
POS IgG CHIKV	8
**Other interfering conditions**	
Pos PCR Malaria	13
POS Rheumatoid Factor	10
**Healthy donors**	
NEG All **	16
Symptomatic samples	
ZIKV IgG negative	93
TOTAL	188

NEG All **: These clinical samples were collected from asymptomatic healthy donors and tested negative by real time-PCR and ELISA IgM/IgG for the following viruses: Rift valley fever (RVF), Crimean Congo hemorrhagic fever (CCHFV), YFV, DENV, WNV, Chikungunya (CHIKV), and ZIKV within the WHO-CC on Arboviruses and Viral Hemorrhagic Fevers of IPD’s virology department.

**Table 2:** Amino acid sequences of E protein domain III of South Pacific strain used for cloning compared to MR766 reference strain.

Viral Strain	Position in E Sequence	EDIII Sequence
**PF-25013-18**	297–341	LRLKGVSYSLCTAAFTFTKIPAETLHGTVTVEVQYAGTDGPCKVP
**MR766**		LRLKGVSYSLCTAAFTFTKIPAETLHGTVTVEVQYAGTDGPCKVP
**PF-25013-18**	342–387	AQMAVDMQTLTPVGRLITANPVITESTENSKMMLELDPPFGDSYIV
**MR766**		VQMAVDMQTLTPVGRLITANPVITESTENSKMMLELDPPFGDSYIV
**PF-25013-18**	388–408	IGVGEKKITHHWHRSGSTIGK
**MR766**		IGVGEKKITHHWHRSGSTIGK

**Table 3:** AUC parameters.

Parameter	Value
Area	0.974
Std. Error	0.009
95% CI	0.974–0.992
*p* value	<0.0001

**Table 4:** Performance characteristics at 0.208.

Parameters	Values
Sensitivity	100.0% [97.4–100.0]
Specificity	94.7% [90.3–97.2]
Likelihood Ratio+	18.8%
Likelihood Ratio−	0%
TP	179
TN	178
FP	10
FN	0
Accuracy	97.3% [95.7–99.2]

**Table 5:** Performance per sample characterization.

Panel Tested	Condition Tested	Reference Test	Analysis Values	Sensitivity	Specificity	*n*	Analyzed Method	Reference Method	
								pos	neg
Sensitivity Panel	ZIKV IgG POS	ZIKV PRNT	estimate:	100.0%	94.7%	367	pos	179	10
			95% CI:	[97.4–100.0]	[90.3–97.2]				
			*p*-value	2.2 × 10^−16^	2.2 × 10^−16^		neg	0	178
Specificity Panel	POS IgG Dengue	ZIKV PRNT	estimate:		95.6%	23	pos		1
			95% CI:		[78.0–99.9]				
			*p*-value		5.72 × 10^−6^		neg		22
	POS IgG WNV	ZIKV PRNT	estimate:		100.0%	9	pos		0
			95% CI:		[66.4–100.0]				
			*p*-value		0.0039		neg		9
	POS IgG YFV	ZIKV PRNT	estimate:		93.7%	16	pos		1
			95% CI:		[69.8–99.8]				
			*p*-value		0.0005		neg		15
	POS IgG CHIKV	ZIKV PRNT	estimate:		100.00%	8	pos		0
			95% CI:		[63.1–100.0]				
			*p*-value		0.007812		neg		8
	POS PCR Malaria	ZIKV PRNT	estimate:		100.0%	13	pos		0
			95% CI:		[75.3–100.0]				
			*p*-value		0.0002		neg		13
	POS Rheumatoid Factor	ZIKV PRNT	estimate:		100.0%	10	pos		0
			95% CI:		[69.1–100.0]				
			*p*-value		0.0020		neg		10
	NEG all **	ZIKV PRNT	estimate:		100.0%	16	pos		0
			95% CI:		[79.4–100.0]				
			*p*-value		3.052 × 10^−5^		neg		16
	Symptomatic IgG Neg ZIKV	ZIKV PRNT	estimate:		91.4%	93	pos		8
			95% CI:		[83.7–96.2]				
			*p*-value		2.2 × 10^−16^		neg		85 **

** NEG all samples were obtained from healthy persons tested negative by RT-PCR and IgG/IgM ELISA for Rift Valley Fever (RVFV), Crimean Congo Haemorrhagic Fever (CCHFV), Yellow Fever (YFV), DENV, West Nile (WNV), Chikungunya (CHIKV) and ZIKV.

**Table 6:** Advantages and drawbacks of EDIII ELISA in comparison to standard IgG ELISA and PRNT.

Type of Test	Advantages	Disadvantages
EDIII IgG ELISA	High sensitivity and specificity Easy to set up High throughput Short “hands-on time” (4 h) Cheap	Semi quantitative
Standard Indirect IgG ELISA	High throughput Short “hands-on time” (5 h)	Low specificity (high cross reactivity) Use of animal tissue (mouse brain) Semi quantitative
PRNT Test	High sensitivity and specificity Measures neutralizing activity Quantitative method	Requires a BSL 3 Lab Requires highly trained personnel Expensive Unable to differentiate antibody classes Slow (4–6 days) to obtain results

## Data Availability

The data presented in this study are available within the article.
